# Antibiotic Sensitivity Screening of *Klebsiella* spp. and *Raoultella* spp. Isolated from Marine Bivalve Molluscs Reveal Presence of CTX-M-Producing *K. pneumoniae*

**DOI:** 10.3390/microorganisms8121909

**Published:** 2020-11-30

**Authors:** Fredrik Håkonsholm, Marit A. K. Hetland, Cecilie S. Svanevik, Arnfinn Sundsfjord, Bjørn Tore Lunestad, Nachiket P. Marathe

**Affiliations:** 1 Institute of Marine Research, P.O. Box 1870 Nordnes, NO-5817 Bergen, Norway; fredrik.haakonsholm@hi.no (F.H.); cecilie.smithsvanevik@hi.no (C.S.S.); bjorn-tore.Lunestad@hi.no (B.T.L.); 2 Department of Medical Biology, Faculty of Health Sciences, University of Tromsø—The Arctic University of Norway, 9037 Tromsø, Norway; arnfinn.sundsfjord@uit.no; 3 Department of Medical Microbiology, Stavanger University Hospital, 4011 Stavanger, Norway; marit.hetland@outlook.com; 4 Department of Biological Sciences, Faculty of Mathematics and Natural Sciences, University of Bergen, 5006 Bergen, Norway; 5 Norwegian National Advisory Unit on Detection of Antimicrobial Resistance, Department of Microbiology and Infection Control, University Hospital of North Norway, 9038 Tromsø, Norway

**Keywords:** *Klebsiella*, bivalve molluscs, antimicrobial resistance, CTX-M

## Abstract

*Klebsiella* spp. are a major cause of both nosocomial and community acquired infections, with *K. pneumoniae* being responsible for most human infections. Although *Klebsiella* spp. are present in a variety of environments, their distribution in the sea and the associated antibiotic resistance is largely unknown. In order to examine prevalence of *K. pneumoniae* and related species in the marine environment, we sampled 476 batches of marine bivalve molluscs collected along the Norwegian coast. From these samples, *K. pneumoniae* (*n* = 78), *K. oxytoca* (*n* = 41), *K. variicola* (*n* = 33), *K. aerogenes* (*n* = 1), *Raoultella ornithinolytica* (*n* = 38) and *R. planticola* (*n* = 13) were isolated. The number of positive samples increased with higher levels of faecal contamination. We found low prevalence of acquired resistance in all isolates, with seven *K. pneumoniae* isolates showing resistance to more than one antibiotic class. The complete genome sequence of cefotaxime-resistant *K. pneumoniae sensu stricto* isolate 2016-1400 was obtained using Oxford Nanopore and Illumina MiSeq based sequencing. The 2016-1400 genome had two contigs, one chromosome of 5,088,943 bp and one plasmid of 191,744 bp and belonged to ST1035. The β-lactamase genes *bla*_CTX-M-3_ and *bla*_TEM-1,_ as well as the heavy metal resistance genes *pco*, *ars* and *sil* were carried on a plasmid highly similar to one found in *K. pneumoniae* strain C17KP0055 from South-Korea recovered from a blood stream infection. The present study demonstrates that *K. pneumoniae* are prevalent in the coastal marine environment and that bivalve molluscs may act as a potential reservoir of extended spectrum β-lactamase (ESBL)-producing *K. pneumoniae* that may be transmitted through the food chain.

## 1. Introduction

The genus *Klebsiella* contains several species known to cause nosocomial infections [1,2] and some that cause community acquired infections [3,4]. *Klebsiella* spp. are widely distributed outside the clinical environment, including environments like soil, plants, surface waters and other mammals [5]. In 2001, *K. terrigena*, *K. ornithinolytica*, *K. planticola* and *K. trevisanii* were assigned to the new genus *Raoultella* [6]. Although considered environmental species, *Raoultella* spp. have gained increased attention as opportunistic pathogens [7].

Within the genus, *K. pneumoniae sensu stricto* is responsible for the majority of human infections [5]. *K. pneumoniae* is closely related to *K. variicola*, *K. quasipneumoniae* subspecies *quasipneumoniae*, *K. quasipneumoniae* subsp. *similipneumoniae*, *K. variicola* subsp. *tropicalensis*, *K. africaensis* and *K. quasivariicola* and together these species constitute the *K. pneumoniae* species complex [8,9,10,11]. Although *Klebsiella* spp. are found in different environments, the opportunistic pathogen *K. pneumoniae* is often present in the human and animal gut [12].

*K. pneumoniae* is considered one of the most important opportunistic pathogens involved in the dissemination of antimicrobial resistance (AMR), as well as one of the most common causes of infections in health care settings [12]. In the EU/EAA countries, 37% of all *K. pneumoniae* isolates reported to the European Antimicrobial Resistance Surveillance Network (EARS-Net) in 2018, had acquired resistance to at least one class of antibiotics [13]. Of special concern is the emergence of carbapenem-resistant *K. pneumoniae* (CR-KP), often also co-resistant to multiple other classes of antibiotics [13]. Resistance to third-generation cephalosporins is a common type of resistance observed in clinical isolates of *K. pneumoniae* in the European countries [13]. This is largely caused by CTX-M-type extended spectrum β-lactamases (ESBLs). CTX-M encoding genes (*bla*_CTX-M_) are often plasmid-borne and spread rapidly among the *Enterobacterales*. Cephalosporin-resistant *Enterobacterales* represents a public health concern as they severely limit the available treatment options [14]. Compared to many other countries, Norway has a low prevalence of AMR and the use of antibacterial agents in both humans and food-producing animals is low [13,15]. However, the prevalence of reported ESBL-producing *K. pneumoniae* in blood stream infections in Norway has increased from 1.9% in 2010 [16] to 8.5% in 2018 [15].

*K. pneumoniae* is genetically a diverse species and the majority of genes are part of the accessory genome [12] which is important in the acquisition of resistance genes [17]. The core genome includes a chromosomal *bla*_SHV_ conferring resistance to aminopenicillins, as well as *oqxAB* and *fosA* mediating reduced susceptibility to quinolones and fosfomycin,, respectively [10]. Most of the acquired AMR genes in *K. pneumoniae* are located on plasmids [18], which can be spread among the members of microbial communities in different environments [19]. It has been suggested that *K. pneumoniae* is a particularly good acceptor of plasmids in diverse ecological niches, with the acquisition of these plasmids having a lower fitness cost in *K. pneumoniae* compared to *Escherichia coli* [12]. Many antibiotic resistance genes (ARGs) originate from the natural environment [20]. The environments affected by anthropogenic activities, for example, wastewater systems and animal manure, are considered hotspots for the development and spread of AMR [21]. However, the role of the marine environment in the development and dissemination of AMR is far from understood. There are multiple transmission routes of ARGs and antibiotic resistant bacteria to the marine environment, for example, sewage and runoff from land [21]. Although the literature is scarce on AMR in opportunistic pathogens in the Norwegian marine environment, previous studies have reported the occurrence of ESBL positive *E. coli* [22,23]. Bivalve molluscs have previously been shown as a good tool for monitoring AMR in the marine environment [22]. As filter feeders, they filter large volumes of water, retain and concentrate particles. As a result, they accumulate high numbers of microorganisms, including bacteria of both aquatic and anthropogenic origin [24]. Bivalve molluscs are therefore good indicators of faecal as well as chemical contamination status in a given marine environment [22,25].

*K. pneumoniae* is extensively studied in clinical settings and some studies have highlighted similarities between clinical and environmental isolates [26,27,28]. However, there is a lack of knowledge on the prevalence of *K. pneumoniae* and related species in the marine environment. The aim of our study was to examine the prevalence of *K. pneumoniae* and related species in the Norwegian marine environment using bivalve molluscs as indicators and study their antibiotic sensitivity patterns.

## 2. Materials and Methods

### 2.1. Sampling

A total of 204 batch samples of bivalve molluscs were collected along the Norwegian coast from September 2019 to March 2020. An additional 272 samples collected in 2016 were included in the study. The samples comprised 384 blue mussels (*Mytilus edulis*), 48 oysters (*Crassostrea gigas*), 24 scallops (*Pecten maximus*), five horse mussels (*Modiolus modiolus*), three ocean quahogs (*Arctica islandica*), two cockles (*Cerastoderma edule*), two carpet shells (*Politapes rhomboides*) and one sand gaper (*Mya arenaria*). Even though not a bivalve mollusc, seven sea urchins (*Strongylocentrotus droebachiensis*) were also included. In total, 476 samples covering 77 different production areas and five non-rearing locations along the Norwegian coast was included in the study. Samples from production areas were collected through the surveillance programme on bivalves conducted by the Norwegian Food Safety Authority (NFSA). A detailed overview of samples and sampling locations is provided in Supplementary Table S1.

### 2.2. Sample Preparation

Each batch sample comprised 10–20 individual bivalves. Live and closed bivalves were cleaned under cold tap water before they were opened using a sterile knife. Approximately 80–100 g soft tissue and intra-valvular fluid was weighed into sterile plastic bags (VWR, Radnor, PA, USA) and homogenised for 2.5 min using a stomacher (Seward, UK).

### 2.3. Isolation and Identification of Presumptive Klebsiella spp.

Aliquots of 25 g were transferred to new sterile plastic bags and diluted 1:10 in Buffered Peptone Water (BPW) (VWR, USA), homogenised for 30 s and incubated aerobically at 37 °C for 18–24 h. After incubation, 10 µL of the enrichment cultures were streaked on Simmons Citrate Agar (Bio-Rad, Hercules, CA, USA) supplemented with 1% Myo-Inositol (Sigma-Aldrich, St. Louis, MO, USA) (SCAI), a highly selective media for the isolation of *Klebsiella* spp. and *Raoultella* spp. [29] and incubated aerobically at 37 °C for 48 h. Samples collected in 2016 had been enriched in BPW by the same protocol and stored at −80 °C in 20% glycerol. Before the samples were analysed, they were thawed in room temperature and approx. 1.5 mL transferred to 10 mL BPW and incubated at 37 °C over night. Yellow colonies representing presumptive *Klebsiella* spp. were sub-cultured to obtain pure cultures. The obtained isolates were cultured overnight on Plate Count Agar (PCA) (Oxoid, UK) at 37 °C. Colonies were transferred directly to disposable 96 spot targets (Bruker, Germany) and covered with 1 µL HCCA matrix (Bruker, Germany). The spots were air dried and the isolates were identified using Matrix Assisted Laser Desorption Time of Flight Mass Spectrometry (MALDI-TOF MS) (Bruker, Germany).

### 2.4. Enumeration of E. coli

Enumeration of *E. coli* was done following ISO16649-3 [30]. The limit of quantification (LOQ) of the method is 18 *E. coli*/100 g samples, hereafter termed < LOQ. Based on the most probable number (MPN) *E. coli*/100 g sample, the sampling sites were categorised as A (<230), B (<4600), C (<46,000) or prohibited (>46,000) for cultivation of bivalves according to EU directive 854/2004 [31].

### 2.5. Antimicrobial Susceptibility Testing

Antimicrobial susceptibility testing (AST) was done by disk diffusion according to the European Committee on Antimicrobial Susceptibility testing (EUCAST) [32]. All isolates were tested against a panel of 17 antimicrobial agents belonging to 10 different classes using antibiotic disks (Oxoid, UK) on Mueller-Hinton agar (MH) (Oxoid, UK). The following agents were included: gentamicin (GEN, 10 µg), chloramphenicol (CHL, 30 µg), meropenem (MEM, 10 µg), cefoxitin (FOX, 30 µg), cefuroxime (CXM, 30 µg), ceftazidime (CAZ, 10 µg), cefotaxime (CTX, 5 µg), aztreonam (ATM, 30 µg), nitrofurantoin (NIT, 100 µg), amoxicillin-clavulanic acid (AMC, 20–10 µg), piperacillin-tazobactam (TZP, 30–6 µg), mecillinam (MEL, 10 µg), ampicillin (AMP, 10 µg), ciprofloxacin (CIP, 5 µg), trimethoprim-sulfamethoxazole (SXT, 1.25–23.75 µg), tetracycline (TET, 30 µg), tigecycline (TGC, 15 µg). *E. coli* CCUG17620 was included as quality control with each set up. The isolates were classified as sensitive (S), intermediate susceptible, increased exposure (I) or resistant (R) according to EUCAST breakpoints for *Enterobacterales* [33]. Breakpoints were unavailable for TET and no inhibition zone was the criterion used for classifying the isolates as resistant. Isolates falling within the area of technical uncertainty (ATU) for TZP were categorised as susceptible, increased exposure (I) to this agent. Isolates resistant to three or more antibiotic classes were defined as multidrug-resistant (MDR) [34].

### 2.6. Whole Genome Sequencing and Sequence Analysis

*K. pneumoniae* isolate 2016-1400 displayed phenotypic resistance to CTX and CXM and was analysed by whole genome sequencing using both short (Illumina) and long reads (Nanopore). For the short read sequencing, DNA was extracted using MagNA Pure 96 and Viral Small volume kit with the Pathogen Universal 200 4.0 purification protocol (Roche Applied Science, Penzberg, Germany). Genomic libraries were prepared using Illumina Nextera DNA Flex library prep and sequenced using the Illumina MiSeq system and the Illumina MiSeq Reagent Kit V3 (600 cycle) to obtain 2 × 300 bp paired end reads. For the long read sequencing, DNA was manually extracted using the Beckman Coulter Life science GenFind V3 kit (C34881) according to the supplemental protocol ‘DNA extraction from Bacteria using GenFind v3′ (Beckman Coulter, Brea, CA, USA). The DNA library was prepared with the Ligation sequencing kit (SQK-LSK109) (Oxford Nanopore Technologies (ONT), Oxford, UK), then loaded onto a R9.4.1 Flongle flow cell (FLO-FLG001) and sequenced on the ONT MinION Mk1B device (MIN-101B). Basecalling was performed with Guppy v4.2.2 + effbaf84 (available to ONT customers at https://community.nanoporetech.com) and quality filtered using FiltLong v0.2.0 (https://github.com/rrwick/Filtlong). Hybrid de novo assembly of the short and long read sequences was performed with Unicycler v0.4.8 [35]. Assembly statistics are available in Supplementary Table S2. The assembled genome was analysed using Kleborate v2.0.0, a tool designed to accurately identify members of the *K. pneumoniae* species complex and sequence types (STs), acquired virulence factors associated with hypervirulent *K. pneumoniae* (yersiniabactin (*ybt*), aerobactin (*iuc*), salmochelin (*iro*) and colibactin (*clb*) as well as the hypermucoidy genes *rmpA*/*mpA2* and acquired ARGs (https://github.com/katholt/Kleborate). Further bioinformatic analysis was done using NCBI Antimicrobial Resistance Gene Finder (AMRFinderPlus) v3.2.3 [36], ABRicate v0.9.8 (https://github.com/tseemann/abricate) using the virulence factors database (VFDB) [37] and PlasmidFinder [38] The genome was annotated using the NCBI Prokaryotic Genome Annotation Pipeline [39]. BLAST Ring Image Generator (BRIG) v0.95 [40] was used for sequence comparison and visualisation. BLASTn v2.9.0 [41] was used to query the *K. pneumoniae* C17KP0055 genome against the *K. pneumoniae* 2016-1400 assembly.

### 2.7. PCR Amplification of bla_SHV_

To confirm the absence of *bla*_SHV_ *K. pneumoniae* isolate 2016-1400 was subjected to PCR amplification of the *bla*_SHV_ gene. DNA was extracted using the DNeasy blood and tissue kit (Qiagen, Hilden, Germany). Each 20 µL reaction contained 4 µL 5X Phusion HF buffer, 0.4 µL 10 mM dNTP mix, 0.2 µL of each 50 µM SHV specific primer (SHVF: 5′-ATGCGTTATATTCGCCTGTG-3′, SHVR: 5′-TGCTTTGTTATTCGGGCCAA-3′) [42], 0.2 µL Phusion DNA polymerase, 1 µL template DNA and 14 µL nuclease free water. PCR amplification was performed using the GeneAmp PCR system 9700 Thermal Cycler (Applied Biosystems, Waltham, MA, USA) and the following conditions: initial denaturation at 98 °C for 30 s, 30 cycles of 98 °C for 5 s, 62 °C for 5 s and 72 °C for 10 s, with a final extension at 72 °C for 3 min. *K. pneumoniae* CCUG 10,785 was included as a positive control and a *bla*_SHV_-negative *E. coli* as a negative control. The PCR products were resolved on a 1% agarose gel stained with Gel Red Nucleic Acid Stain (Biotium, Fremont, CA, USA) and visualised on a Bio-Rad ChemiDoc system (Bio-Rad, USA).

### 2.8. Filter Conjugation

Conjugation was carried out according to the method described by Jutkina et al., 2016 [43]. Briefly, kanamycin (KAN) and rifampicin (RIF) resistant *gfp* marked *E. coli* recipient was grown in Mueller Hinton broth (MHB) (Oxoid, UK) with 50 µg/mL KAN (Sigma-Aldrich, St. Louis, MO, USA) at 30 °C with shaking overnight. The CTX and AMP resistant donor was grown over night in MHB supplemented with 2 µg/mL CTX (Sigma-Aldrich, St. Louis, MO, USA) under the same incubation conditions. The donor and recipient were centrifuged at 2755× *g* for 15 min. and washed twice in phosphate buffered saline (PBS) (Sigma-Aldrich, St. Louis, MO, USA) before final resuspension in PBS. The conjugation mixtures were prepared by mixing equal aliquots of donor and recipients (1:1 ratio). The conjugation mixture was pipetted on to 0.45 µm filters (Merck Millipore, Burlington, MA, USA) and placed on Mueller Hinton (MH) (Oxoid, UK) agar plates and incubated at 37 °C overnight. After incubation, the filter was removed and placed in a falcon tube with 10 mL PBS and sterile glass beads and the cells were removed from the filter by vortexing at maximum speed for 90 s. Serial dilutions up to 10^−6^ was prepared in PBS and 100 µL spread in duplicates on CHROMagar orientation (CHROMagar, Paris, France) plates supplemented with 50 µg/mL KAN, 50 g/mL RIF and 100 µg/mL AMP (Sigma-Aldrich, St. Louis, MO, USA) and 50 µg/mL KAN, 50 µg/mL RIF and 2 µg/mL CTX. The plates were incubated at 37 °C for 24–30 h.

## 3. Results

### 3.1. Distribution of Klebsiella spp. and Raoultella spp. in Marine Bivalves

From the 476 samples, presumptive *Klebsiella* spp. were detected in 41% (*n* = 194) of the samples, with some samples positive for several morphotypes. A total of 204 isolates were obtained and identified as members of the genera *Klebsiella* and *Raoultella* using MALDI-TOF MS. In total, 78 isolates were identified as *K. pneumoniae*, 41 as *K. oxytoca*, 33 *K. variicola*, one *K. aerogenes*, 38 as *R. ornithinolytica* and 13 isolates were identified as *R. planticola* (Table 1).

**Figure 1:**
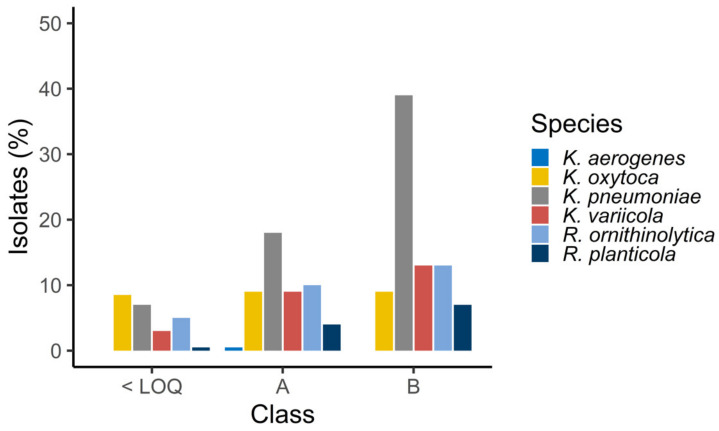
Distribution (%) of *Klebsiella* spp. and *Raoultella* spp. isolates recovered from areas of increasing *E. coli* load according to the EU classification A, B and C. < LOQ: < 18 MPN *E. coli*/100 g, A: 18-230 MPN *E. coli*/100 g, B: 230-4600 MPN *E. coli*/100 g. No results corresponded to *E. coli* counts above B classification.

The frequency of samples positive for *Klebsiella* spp. and/or *Raoultella* spp. increased with higher levels of faecal contamination as expressed by the number of *E. coli* detected, with a total of 24% (*n* = 51), 48% (*n* = 96) and 81% (*n* = 56) of the samples positive from < LOQ (*n* = 213) areas, class A (*n* = 194) areas and class B (*n* = 69) areas, respectively. The most frequently isolated species from class A and B areas was *K. pneumoniae* and *K. oxytoca* from locations where *E. coli* MPN/100 g was < LOQ (Figure 1). Detailed overview of isolates is provided in Supplementary Table S3.

### 3.2. Antimicrobial Susceptibility Patterns of Klebsiella spp. and Raoultella spp.

Among the *K. pneumoniae* isolates, resistance to more than one agent was seen in only eight isolates. Three MDR isolates were detected (Supplementary Table S4), while one isolate displayed phenotypic resistance to cefuroxime, cefotaxime and ampicillin, as well as intermediate susceptibility to aztreonam.

Phenotypic ampicillin susceptibility was observed in *K. pneumoniae* (5%, *n* = 4), *K. oxytoca* (5%, *n* = 2), *K. variicola* (21%, *n* = 7) and *R. ornithinolytica* (8%, *n* = 3) in repeated experiments. (Table 2). Measured antibiotic inhibition zones of all isolates are included in Supplementary Table S4.

### 3.3. Genome Sequencing

The sequenced genome of *K. pneumoniae sensu stricto* isolate 2016-1400 was de novo assembled into two contigs, one 5,088,943 bp chromosome and one 191,744 bp plasmid. The isolate belonged to ST1035 and had the *wzi* allele 116, corresponding to capsule locus (KL) type 57. Further analysis of the sequenced genome revealed that the isolate carried *bla*_CTX-M-3_, *bla*_TEM-1_, *oqxA*, *oqxB*, *fosA* and *erm(D)* conferring resistance to erythromycin but lacked the *bla*_SHV_ gene. This was confirmed by PCR analysis. A comparison of 2016-1400 with SHV-1-harbouring *K. pneumoniae* ST1035 genomes (ENA run accession number ERR4859177 and ERR3416161) showed that there had likely been a deletion of a 10.1 Kbp region, which included *bla*_SHV-1_, due to the insertion of an IS5 family transposase between a hypothetical protein and diguanylate phosphodiesterase in our isolate.

Several heavy metal resistance genes, including the plasmid borne copper resistance system(*pco*) gene cluster, the arsenic resistance genes (*ars*) and the *sil* operon genes conferring resistance to silver were identified. Virulence genes involved in iron acquisition (*ent* and *fep*), adherence (*ecp*), magnesium uptake (*mgt*) and immune evasion (*ompA*) were also detected in 2016-1400 (Table 3).

**Figure 2:**
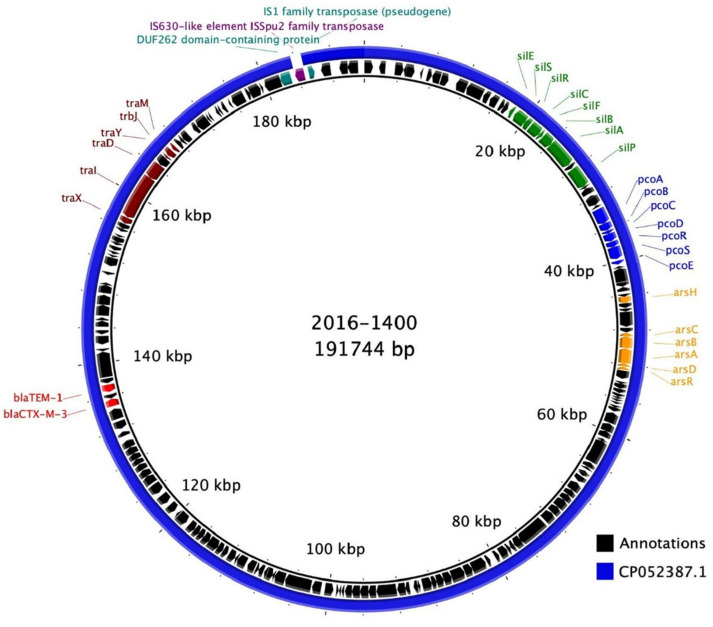
BLAST Ring Image Generator (BRIG) comparison of the *K. pneumoniae sensu stricto* 2016-1400 plasmid (accession number CP065035) and the plasmid from *K. pneumoniae* strain C17KP0055 (accession number CP052387.1). Ring 1 (innermost) shows the positions in the 2016-1400 plasmid, ring 2 shows its gene annotations and ring 3 shows the BLASTn result of CP052387.1 against the 2016-1400 plasmid. The locations of *bla*_CTX-M-3_, *bla*_TEM-1_, the *sil*, *pco*, *ars* operons and *tra* genes are indicated. One gene, an IS630-like element ISSpu2 family transposase, was not present in CP052387.1.

*bla*_CTX-M-3_ and *bla*_TEM-1_, as well as the heavy metal resistance genes were carried on a IncFIB(K)/IncFII plasmid highly similar to the190 582 bp non-conjugstiveCP052387.1 IncFIB(K)/IncFII plasmid (100% sequence coverage and 99.96% nucleotide identity) from a clinical *K. pneumoniae* strain (Figure 2). The plasmid was not transferable by filter conjugation and carried few conjugal transfer genes. These plasmids carried all of the same genes, except an IS630-like element ISSpu2 family transposase, which was carried by 2016-1400 only. Compared to CP052387.1, this insertion occurred between two base pairs in a pseudogene, an incomplete hypothetical protein. The 5,083,236 bp chromosome of the same published genome (CP052386.1) was also similar to 2016-1400, with 99.62% sequence coverage and 99.43% nucleotide identity.

## 4. Discussion

Here we present a comprehensive study on the prevalence of *K. pneumoniae* and related species in marine bivalves collected from both areas used for commercial production of bivalve molluscs for human consumption along the Norwegian coast as well as non-rearing locations in Western and Southern Norway. We further show low prevalence of acquired antibiotic resistance in these isolates. However, one *K. pneumoniae* isolate carried a clinically important and potentially mobile ESBL gene.

*K. pneumoniae sensu stricto* isolate 2016-1400 recovered from *M. edulis* at a production area in Middle-Norway displayed phenotypic resistance to cefotaxime and carried a plasmid encoding the *bla*_CTX-M-3_ gene, first described in clinical *E. coli* and *Citrobacter freundii* isolates in Poland in 1996 [44]. The CTX-M genes originated from *Kluyvera* spp. and were spread through mobilisation from their chromosomal position [14,45] and since they have disseminated worldwide [14]. Cephalosporins are among the most commonly used classes of antibiotics worldwide [46] and ESBL-producing *K. pneumoniae* represents a threat to the public health as it limits the available therapeutic options [47]. The plasmid also carried a *bla*_TEM-1_ gene, a common plasmid-borne resistance gene among clinical Gram-negative bacteria, primarily conferring resistance to penicillins and first- generation cephalosporins [48]. No gene encoding the chromosomal *bla*_SHV_ was identified in *K. pneumoniae* 2016-1400. Interestingly, no chromosomal *bla*_SHV_ was detected in the chromosome (CP052386.1) of *K. pneumoniae* strain C17KP0055 from South-Korea, which is highly similar to the strain in our study. This is rare but absence of SHV has previously been reported in some *K. pneumoniae* strains [49,50].

A recent study on antibiotic resistance in *E. coli* from marine bivalves collected in Norway identified *bla*_CTX-M_ genes (*bla*_CTX-M-15_ and *bla*_CTX-M-14_) in only 1% of the isolates [22]. This is in line with our findings, indicating a low prevalence of CTX-M- producing *Enterobacterales* in the Norwegian marine environment. Although the occurrence of ESBL-producing *Enterobacterales* in Norway is low compared to many other countries, the prevalence of clinical ESBL-producing *Klebsiella* spp. isolates is increasing [15]. Hence, the presence of ESBL-producing *Enterobacterales* in reared bivalves intended for human consumption is concerning.

Furthermore, the *K. pneumoniae* 2016-1400 plasmid carried genes conferring resistance to silver, copper and arsenic. The plasmid was highly similar to the plasmid of a *K. pneumoniae* strain (C17KP0055) isolated from the blood of a South-Korean patient, encoding the indistinguishable ARGs and heavy metal resistance genes. Co-localisation of heavy metal resistance genes and ARGs on the same mobile genetic element (MGE) increases the chance of co-selection by heavy metals [51]. High copper and arsenic levels have been detected in some marine environments in Norway [52] and this may contribute to the co-selection of this plasmid/strain.

*K. pneumoniae* ST1035 has been associated with human infections [53,54,55,56]. Furthermore, capsule type (K) 57 has been identified in clinical isolates and associated with pyogenic liver abscess (PLA) [57,58]. However, isolate 2016-1400 did not harbour any of the genes associated with hypervirulent *K. pneumoniae* (hvKp) or the capsule types (K1 and K2) which are mainly associated with hvKP. Analysis of *K. pneumoniae* strain C17KP0055 showed that this strain belonged to the same ST and did not harbour any of the hvKP associated virulence genes.

One MDR *K. pneumoniae* isolate (2019-1764) was recovered from a location close to the city centre of Bergen. The location is likely to be heavily influenced by anthropogenic activities, indicating that this isolate is of human or animal origin. This could pose a threat to the public health as the sampling location is in close proximity to bathing areas [59].

The overall results from our study are in accordance with previous studies on AMR in bacteria from the Norwegian marine environment, where low prevalence of acquired resistance was found in *E. coli* and *Vibrio* spp. [22,60,61]. In the present study, acquired resistance was only found in few *K. pneumoniae* isolates and in none of the other isolated species. These results reflect the restrictive use of antibiotics in Norway, both in clinics, food production and companion animals. In Norwegian clinical *K. pneumoniae* isolates, resistance to amoxicillin-clavulanic acid, trimethoprim, trimethoprim-sulfamethoxazole, cefuroxime and mecillinam are the most commonly observed resistance phenotypes [15]. Resistance to these agents, except mecillinam, was also found among the marine *K. pneumoniae* isolates. No carbapenemase-producing isolates were recovered. This is in contrast to the increase of CR-KP in some European countries [13] as well as several reports on the occurrence of CR-KP in the environment [59,62,63,64]. Although all of the isolated species are considered to be intrinsically resistant to aminopenicillins due to the presence of chromosomal class A β-lactamases [5,65], we found some ampicillin-susceptible isolates among all species, except *K. aerogenes* and *R. planticola*. This has previously been described in *Klebsiella* spp. isolates [66,67,68,69] and is in *K. pneumoniae* likely caused by differential expression [70] or lack of the *bla*_SHV_ gene.

*K. pneumoniae* was the most common species isolated from our samples. This is in accordance with a study by Podschun et al. [28] on *Klebsiella* spp. in surface waters from fresh, brackish and seawater in temperate regions. The presence of *K. oxytoca* seems to be less dependent on faecal contamination than *K. pneumoniae*. As *K. oxytoca* is known to be present in terrestrial environments [71], there are several transmission routes for this species to the marine environment. The relative distribution of the isolated genera in our study is in contradiction to the current description of the genus *Raoultella* as an environmental genus known to be associated with aquatic environments [6,65,72]. Even from samples with *E. coli* MPN/100 g below the LOQ, *Klebsiella* was the more frequently isolated compared to *Raoultella*.

This study provides enhanced understanding about the prevalence and AMR of *K. pneumoniae* found in the Norwegian marine environment. Although low prevalence of acquired resistance was detected, our study demonstrates presence of ESBL-producing *K. pneumoniae* in an area used for production of marine bivalves for human consumption. This indicates that bivalve molluscs may act as a potential reservoir of ESBL-producing *K. pneumoniae* for transmission to the community through the food chain. Our study also highlights the importance of the marine environment in dissemination of opportunistic human pathogens and ARGs.

## Figures and Tables

**Table 1:** *Klebsiella* spp. and *Raoultella* spp. isolated from different bivalve mollusc species, *n* in brackets refer to the number of examined samples for each bivalve species

	Bivalve Species				
Species	*M. edulis* (*n* = 384)	*P. maximus* (*n* = 24)	*C. gigas* (*n* = 48)	*P. rhomboides* (*n* = 2)	Total No. Isolates
*K. pneumoniae*	70	2	5	1	78
*K. oxytoca*	40	0	1	0	41
*K. variicola*	29	1	3	0	33
*K. aerogenes*	1	0	0	0	1
*R. ornithinolytica*	25	5	8	0	38
*R. planticola*	12	0	1	0	13

**Table 2:** Prevalence of antibiotic resistance among *Klebsiella* spp. and *Raoultella* spp. isolated form marine bivalves.

							Antibacterial Agent										
Species	AMP	MEL	AMC	TZP	CHL	GEN	CIP	NIT	SXT	TET	TGC	CTX	CAZ	FOX	CXM	ATM	MEM
*K. pneumoniae* (*n* = 78)	74	0	3	3 *	2	0	1	2	2	3	0	1	0	0	1	1 *	0
*K. oxytoca* (*n* = 41)	39	0	0	0	0	0	0	0	0	0	0	0	0	0	0	0	0
*K. variicola* (*n* = 33)	26	0	0	0	0	0	0	0	0	0	0	0	0	0	0	0	0
*K. aerogenes* (*n* = 1)	1	0	1	0	0	0	0	0	0	0	0	0	0	1	0	0	0
*R. ornithinolytica* (*n* = 38)	35	0	0	0	0	0	0	0	0	0	0	0	0	0	0	0	0
*R. planticola* (*n* = 13)	13	0	0	0	0	0	0	0	0	0	0	0	0	0	0	0	0

* Isolates categorised as intermediate susceptible, increased exposure. Abbreviations: AMP: Ampicillin, MEL: Mecillinam, AMC: Amoxicillin-clavulanic acid, TZP: Piperacillin-Tazobactam, CHL: Chloramphenicol, GEN: Gentamicin, CIP: Ciprofloxacin, NIT: Nitrofurantoin, SXT: Trimethoprim-sulfamethoxazole, TET: Tetracycline, TGC: Tigecycline, CTX: Cefotaxime, CAZ: Ceftazidime, FOX: Cefoxitin, CXM: Cefuroxime, ATM: Aztreonam, MEM: Meropenem.

**Table 3:** Genes for antibiotic resistance, heavy metal resistance and virulence identified in *K. pneumoniae* isolate 2016-1400.

*Isolate*	*ARGs*	*HRGs*	*VGs*	*Accession nos.*
*2016-1400*	*bla*_CTX-M-3_, *bla*_TEM-1_, *oqxA*, *oqxB*, *fosA*, *erm(D)*	*pcoA*, *pcoB*, *pcoC*, *pcoD*, *pcoR*, *pcoS*, *pcoE*, *arsB*, *arsA*, *arsD*, *arsR*, *arsC*, *arsH*, *SilE*, *SilS*, *SilR*, *SilC*, *SilF*, *SilB*, *SilA*, *SilP*	*ent*, *fep*, *ecp*, *mgt*, *ompA*	CP065034, CP065035

Abbreviations: ARGs: Antibiotic resistance genes, HRGs: Heavy metal resistance genes, VGs: Virulence genes.

## Data Availability

The genome sequence included in the study has been submitted to GenBank with the accession numbers CP065034 (chromosome) and CP065035 (plasmid). The short reads (ERR4570363) and long reads (ERR4859178) are available under BioProject PRJEB40149 in the European Nucleotide Archive.

## Supplementary Materials

The following are available online at https://www.mdpi.com/2076-2607/8/12/1909/s1, Table S1: Sample origin, bivalve species and *E. coli*/MPN 100 g, Table S2: Assembly statistics for whole genome sequence of *K. pneumoniae* isolate 2016-1400, Table S3: Species identification by MALDI-TOF-MS, isolate origin and *E. coli*/MPN 100 g, Table S4: Measured inhibition zones (mm) from AST of *Klebsiella* spp. and *Raoultella* spp.
