# Supplementary material for: Antibiotic Sensitivity Screening of Klebsiella spp. and Raoultella spp. Isolated from Marine Bivalve Molluscs Reveal Presence of CTX-M-Producing K. pneumoniae
Source: Microorganisms. 2020 Nov 30;8(12):1909. doi: 10.3390/microorganisms8121909 (PMC7761178; doi:10.3390/microorganisms8121909)
Supplement: Supplementary file 1 [file microorganisms-08-01909-s001.zip › Table S2.docx]

Table S2: Assembly statistics for whole genome sequence of *K. pneumoniae* isolate 2016-1400

| **Isolate ID** | **Species** | **Chromosome accession no.** | **Plasmid accession no.** | **Tot. Length** | **No. Contigs** | **GC (%)** | **N50** | **CDSs (Total)** |
| --- | --- | --- | --- | --- | --- | --- | --- | --- |
| 2016-1400 | *K. pneumoniae* | CP065034 | CP065035 | 5280687 | 2 | 57.39 | 5088943 | 5025 |

Abbreviations: CDSs: Coding sequences.
