# Supplementary material for: Antibiotic Sensitivity Screening of Klebsiella spp. and Raoultella spp. Isolated from Marine Bivalve Molluscs Reveal Presence of CTX-M-Producing K. pneumoniae
Source: Microorganisms. 2020 Nov 30;8(12):1909. doi: 10.3390/microorganisms8121909 (PMC7761178; doi:10.3390/microorganisms8121909)
Supplement: Supplementary file 1 [file microorganisms-08-01909-s001.zip › Table S4.docx]

Table S4: Measured inhibition zones (mm) from AST of *Klebsiella*spp. and *Raoultella* spp.

| **Antibacterial agent. Inhibition zone (mm)** | | | | | | | | | | | | | | | | | | |
| --- | --- | --- | --- | --- | --- | --- | --- | --- | --- | --- | --- | --- | --- | --- | --- | --- | --- | --- |
| **Species** | **Isolate ID** | **AMP** | **MEL** | **AMC** | **TZP** | **CHL** | **GEN** | **CIP** | **NIT** | **SXT** | **TET** | **TGC** | **CTX** | **CAZ** | **FOX** | **CXM** | **ATM** | **MEM** |
| *K. pneumoniae* | 2016-1072 | 0 | 27 | 25 | 23 | 28 | 20 | 33/28 | 15 | 26 | 23 | 20 | 26 | 26 | 25 | 24 | 33 | 32 |
| *K. pneumoniae* | 2016-1076 | 13 | 28 | 24 | 28 | 29 | 21 | 34 | 16 | 32 | 26 | 23 | 29 | 29 | 26 | 28 | 34 | 31 |
| *K. pneumoniae* | 2016-1145 | 16 | 31 | 27 | 28 | 30 | 21 | 34 | 27 | 32 | 26 | 21 | 32 | 30 | 27 | 28 | 34 | 31 |
| *K. pneumoniae* | 2016-1178 | 17 | 30 | 26 | 26 | 28 | 21 | 32 | 20 | 30 | 26 | 22 | 30 | 27 | 26 | 28 | 34 | 32 |
| *K. pneumoniae* | 2016-1198 | 0 | 25 | 24 | 23 | 0 | 20 | 29 | 16 | 22 | 0 | 21 | 28 | 27 | 26 | 24 | 33 | 32 |
| *K. pneumoniae* | 2016-1200 | 0 | 21 | 19 | 21 | 26 | 20 | 31 | 18 | 0 | 0 | 22 | 29 | 29 | 27 | 26 | 33 | 33 |
| *K. pneumoniae* | 2016-1201 | 12 | 28 | 24 | 23 | 28 | 20 | 31 | 22 | 30 | 24 | 22 | 28 | 28 | 25 | 24 | 33 | 31 |
| *K. pneumoniae* | 2016-1214 | 0 | 27 | 24 | 27 | 24 | 18 | 28 | 16 | 29 | 21 | 19 | 30 | 28 | 24 | 24 | 34 | 34 |
| *K. pneumoniae* | 2016-1241 | 0 | 24 | 24 | 23 | 25 | 20 | 27 | 13 | 27 | 21 | 20 | 28 | 28 | 25 | 26 | 34 | 34 |
| *K. pneumoniae* | 2016-1245 | 8 | 27 | 26 | 24 | 27 | 21 | 28 | 16 | 26 | 23 | 19 | 28 | 27 | 26 | 25 | 35 | 32 |
| *K. pneumoniae* | 2016-1246 | 8 | 25 | 24 | 22 | 27 | 20 | 28 | 15 | 25 | 22 | 21 | 28 | 27 | 25 | 24 | 30 | 29 |
| *K. pneumoniae* | 2016-1248 | 0 | 25 | 25 | 22 | 26 | 20 | 32 | 16 | 29 | 24 | 20 | 28 | 27 | 24 | 23 | 31 | 30 |
| *K. pneumoniae* | 2016-1249 | 10 | 29 | 26 | 24 | 28 | 21 | 30 | 15 | 27 | 24 | 21 | 30 | 27 | 26 | 25 | 34 | 31 |
| *K. pneumoniae* | 2016-1292 | 0 | 27 | 25 | 23 | 26 | 21 | 29 | 21 | 27 | 23 | 19/20 | 28 | 28 | 25 | 24 | 34 | 31 |
| *K. pneumoniae* | 2016-1331 | 12 | 28 | 25 | 22 | 27 | 20 | 30 | 19 | 28 | 24 | 20 | 27 | 27 | 24 | 24 | 32 | 30 |
| *K. pneumoniae* | 2016-1332 | 0 | 28 | 24 | 21 | 25 | 20 | 27 | 19 | 25 | 21 | 19 | 29 | 28 | 24 | 23 | 34 | 34 |
| *K. pneumoniae* | 2016-1394 | 10 | 28 | 26 | 23 | 27 | 21 | 29 | 18 | 26 | 23 | 20 | 28 | 26 | 25 | 24 | 31 | 30 |
| *K. pneumoniae* | 2016-1395 | 8 | 26 | 24 | 22 | 25 | 19 | 29 | 18 | 26 | 23 | 20 | 26 | 26 | 25 | 24 | 34 | 30 |
| *K. pneumoniae* | 2016-1396 | 10 | 29 | 25 | 24 | 27 | 20 | 30 | 21 | 26 | 23 | 19 | 29 | 28 | 26 | 26 | 35 | 32 |
| *K. pneumoniae* | 2016-1397 | 18 | 30 | 27 | 30 | 30 | 21 | 32 | 19 | 30 | 25 | 21 | 31 | 30 | 26 | 28 | 35 | 31 |
| *K. pneumoniae* | 2016-1398 | 0 | 26 | 24 | 21 | 25 | 19 | 30 | 15 | 28 | 23 | 21 | 27 | 26 | 25 | 24 | 32 | 30 |
| *K. pneumoniae* | 2016-1399 | 9 | 28 | 26 | 24 | 26 | 20 | 28 | 15 | 27 | 23 | 20 | 27 | 28 | 26 | 24 | 32 | 31 |
| *K. pneumoniae* | 2016-1400 | 0 | 23 | 20 | 22 | 26 | 20 | 28 | 16 | 26 | 23 | 19 | 13 | 24 | 26 | 0 | 25 | 32 |
| *K. pneumoniae* | 2016-201 | 0 | 27 | 25 | 20 | 25 | 21 | 32 | 19 | 28 | 24 | 20 | 27 | 26 | 23 | 21 | 32 | 32 |
| *K. pneumoniae* | 2016-264 | 0 | 28 | 25 | 23 | 25 | 22 | 28 | 21 | 26 | 22 | 19 | 27 | 26 | 22 | 23 | 30 | 31 |
| *K. pneumoniae* | 2016-319 | 0 | 23 | 18 | 19 | 27 | 21 | 29 | 14 | 27 | 0 | 19 | 28 | 26 | 24 | 24 | 32 | 31 |
| *K. pneumoniae* | 2016-498 | 0 | 29 | 24 | 22 | 26 | 20 | 31 | 15 | 28 | 23 | 20 | 29 | 28 | 24 | 24 | 32 | 33 |
| *K. pneumoniae* | 2016-563 | 0 | 28 | 25 | 22 | 24 | 20 | 31 | 11 | 28 | 23 | 20 | 30 | 29 | 24 | 25 | 34 | 34 |
| *K. pneumoniae* | 2016-564 | 0 | 29 | 25 | 23 | 25 | 20 | 32 | 10 | 29 | 23 | 20 | 29 | 27 | 25 | 25 | 34 | 30 |
| *K. pneumoniae* | 2016-566 | 8 | 27 | 26 | 23 | 27 | 21 | 29 | 15 | 26 | 23 | 20 | 29 | 26 | 25 | 25 | 32 | 30 |
| *K. pneumoniae* | 2016-567 | 0 | 25 | 23 | 20 | 24 | 20 | 27/30 | 16 | 28 | 23 | 21 | 28 | 25 | 23 | 22 | 31 | 30 |
| *K. pneumoniae* | 2016-637 | 0 | 27 | 24 | 21 | 25 | 19 | 30 | 17 | 28 | 22 | 19 | 27 | 26 | 24 | 23 | 32 | 31 |
| *K. pneumoniae* | 2016-681 | 0 | 27 | 24 | 22 | 26 | 20 | 29 | 20 | 28 | 22 | 19 | 29 | 28 | 23 | 24 | 32 | 32 |
| *K. pneumoniae* | 2016-727 | 0 | 28 | 25 | 20 | 26 | 21 | 29 | 21 | 26 | 21 | 19 | 30 | 28 | 24 | 23 | 33 | 34 |
| *K. pneumoniae* | 2016-729 | 0 | 28 | 24 | 22 | 26 | 20 | 28 | 19 | 30 | 22 | 19 | 27 | 26 | 22 | 22 | 31 | 32 |
| *K. pneumoniae* | 2016-733 | 0 | 26 | 24 | 21 | 26 | 21 | 29 | 16 | 27 | 23 | 20 | 28 | 27 | 24 | 23 | 33 | 32 |
| *K. pneumoniae* | 2016-734 | 0 | 28 | 25 | 21 | 26 | 21 | 31 | 19 | 28 | 24 | 19 | 28 | 28 | 25 | 24 | 34 | 34 |
| *K. pneumoniae* | 2016-735 | 0 | 27 | 24 | 20 | 26 | 20 | 29 | 18 | 27 | 24 | 20 | 27 | 27 | 25 | 23 | 34 | 32 |
| *K. pneumoniae* | 2016-833 | 0 | 25 | 23 | 19 | 24 | 20 | 27/30 | 21 | 25/27 | 20 | 18/20 | 28 | 26 | 22/24 | 22 | 33 | 32 |
| *K. pneumoniae* | 2016-873 | 0 | 29 | 25 | 22 | 26 | 22 | 32 | 20 | 27 | 24 | 21 | 28 | 26 | 23 | 23 | 31 | 31 |
| *K. pneumoniae* | 2016-921 | 0 | 27 | 23 | 23 | 27 | 20 | 30 | 24 | 30 | 21 | 19 | 29 | 28 | 23 | 24 | 35 | 33 |
| *K. pneumoniae* | 2019-1394 | 0 | 27 | 25 | 23 | 25 | 19 | 31 | 12 | 28 | 26 | 21 | 29 | 28 | 27 | 25 | 33 | 31 |
| *K. pneumoniae* | 2019-1396 | 0 | 28 | 25 | 21 | 26 | 21 | 30 | 19 | 26 | 25 | 21 | 27 | 27 | 25 | 23 | 34 | 34 |
| *K. pneumoniae* | 2019-1417/2 | 0 | 25 | 24 | 21 | 25 | 19 | 28 | 16 | 27 | 23 | 20 | 28 | 27 | 25 | 23 | 32 | 30 |
| *K. pneumoniae* | 2019-1434 | 9 | 28 | 25 | 24 | 26 | 20 | 33 | 22 | 29 | 25 | 22 | 29 | 27 | 25 | 25 | 34 | 32 |
| *K. pneumoniae* | 2019-1435 /2 | 0 | 26 | 23 | 21 | 25 | 20 | 31 | 21 | 27 | 22 | 19 | 29 | 25 | 24 | 22 | 31 | 31 |
| *K. pneumoniae* | 2019-1444 | 0 | 28 | 25 | 23 | 26 | 20 | 30 | 20 | 28 | 24 | 20 | 29 | 27 | 25 | 24 | 32 | 30 |
| *K. pneumoniae* | 2019-1459 | 0 | 27 | 25 | 22 | 26 | 21 | 30 | 20 | 28 | 24 | 20 | 29 | 27 | 25 | 25 | 33 | 33 |
| *K. pneumoniae* | 2019-1493 /2 | 0 | 28 | 24 | 22 | 26 | 20 | 30 | 18 | 27 | 25 | 20 | 28 | 30 | 26 | 25 | 33 | 32 |
| *K. pneumoniae* | 2019-1496 | 0 | 29 | 26 | 23 | 27 | 21 | 29 | 17 | 28 | 23 | 20 | 27 | 26 | 24 | 22 | 33 | 31 |
| *K. pneumoniae* | 2019-1497 | 0 | 28 | 25 | 22 | 26 | 21 | 30 | 18 | 29 | 25 | 20 | 29 | 27 | 25 | 24 | 34 | 32 |
| *K. pneumoniae* | 2019-1499 | 0 | 25 | 25 | 22 | 25 | 20 | 28 | 13 | 27 | 23 | 19 | 28 | 26 | 26 | 24 | 33 | 31 |
| *K. pneumoniae* | 2019-1718 | 0 | 31 | 27 | 25 | 29 | 21 | 33 | 21 | 29 | 25 | 21 | 30 | 30 | 24 | 23 | 34 | 33 |
| *K. pneumoniae* | 2019-1764 | 0 | 21 | 18 | 21 | 10/0 | 20 | 20 | 14 | 0 | 23 | 20 | 27 | 26 | 24 | 23 | 32 | 30 |
| *K. pneumoniae* | 2019-1791 /1 | 0 | 30 | 25 | 22 | 28 | 21 | 32 | 23 | 28 | 25 | 20 | 30 | 28 | 25 | 25 | 33 | 31 |
| *K. pneumoniae* | 2019-1792 | 0 | 27 | 24 | 21 | 25 | 20 | 30 | 16 | 31 | 9 | 20 | 29 | 28 | 24 | 25 | 33 | 31 |
| *K. pneumoniae* | 2019-1814 | 0 | 25 | 25 | 22 | 25 | 20 | 28 | 14 | 28 | 24 | 19 | 29 | 29 | 26 | 25 | 33 | 32 |
| *K. pneumoniae* | 2019-1897 | 0 | 29 | 26 | 24 | 30 | 20 | 30 | 10/11 | 27 | 26 | 21 | 29 | 26 | 28 | 25 | 33 | 30 |
| *K. pneumoniae* | 2019-1898 | 0 | 28 | 25 | 23 | 27 | 19 | 33 | 15 | 29 | 25 | 19 | 29 | 28 | 25 | 24 | 36 | 30 |
| *K. pneumoniae* | 2019-1903 | 13 | 31 | 26 | 28 | 30 | 20 | 32 | 20 | 27 | 25 | 22 | 30 | 30 | 26 | 28 | 36 | 29 |
| *K. pneumoniae* | 2019-2010 | 0 | 22 | 18 | 18 | 28 | 19 | 31 | 19 | 31 | 25 | 21 | 30 | 27 | 26 | 25 | 33 | 32 |
| *K. pneumoniae* | 2019-2011 /1 | 9 | 29 | 26 | 21 | 27 | 20 | 30 | 18 | 29 | 27 | 20 | 29 | 27 | 26 | 23 | 34 | 31 |
| *K. pneumoniae* | 2019-2017 /2 | 0 | 26 | 26 | 24 | 27 | 20 | 32 | 16 | 26 | 25 | 20 | 28 | 29 | 26 | 26 | 34 | 32 |
| *K. pneumoniae* | 2019-2060 | 0 | 29 | 27 | 24 | 27 | 20 | 30 | 21 | 26 | 24 | 20 | 29 | 26 | 24 | 24 | 35 | 32 |
| *K. pneumoniae* | 2019-2063 /2 | 0 | 31 | 27 | 24 | 28 | 22 | 33 | 22 | 29 | 26 | 22 | 31 | 28 | 25 | 24 | 36 | 33 |
| *K. pneumoniae* | 2019-2120 | 13 | 30 | 26 | 27 | 30 | 20 | 34 | 17 | 30 | 27 | 20 | 31 | 31 | 28 | 29 | 36 | 31 |
| *K. pneumoniae* | 2019-2186 | 0 | 30 | 27 | 25 | 28 | 21 | 29 | 15 | 27 | 23 | 19 | 29 | 27 | 25 | 24 | 33 | 32 |
| *K. pneumoniae* | 2019-2196 | 0 | 27 | 26 | 24 | 28 | 19 | 29 | 16 | 27 | 24 | 19 | 28 | 28 | 25 | 23/24 | 32 | 31 |
| *K. pneumoniae* | 2019-2198 | 0 | 29 | 27 | 21 | 25 | 19 | 27 | 20 | 28 | 23 | 18 | 29 | 28 | 25 | 23 | 33 | 35 |
| *K. pneumoniae* | 2019-2231 | 0 | 27 | 24 | 23 | 27 | 20 | 31 | 17 | 30 | 25 | 21 | 29 | 27 | 24 | 24 | 32 | 32 |
| *K. pneumoniae* | 2019-2233 | 0 | 27 | 24 | 23 | 26 | 18 | 30 | 21 | 34 | 25 | 19 | 29 | 27 | 24 | 25 | 33 | 29 |
| *K. pneumoniae* | 2019-2238 | 17 | 29 | 27 | 30 | 32 | 19 | 32 | 18 | 29 | 26 | 20 | 28 | 27 | 24 | 27 | 32 | 28 |
| *K. pneumoniae* | 2020-114 | 0 | 30 | 27 | 24 | 28 | 20 | 31 | 24 | 30 | 24 | 19 | 30 | 29 | 25 | 25 | 34 | 33 |
| *K. pneumoniae* | 2020-312 /2 | 12 | 31 | 25 | 25 | 29 | 21 | 34 | 21 | 30 | 24 | 20 | 29 | 29 | 26 | 25 | 36 | 33 |
| *K. pneumoniae* | 2020-314 | 0 | 28 | 25 | 21 | 27 | 21 | 30 | 20 | 32 | 25 | 21 | 30 | 28 | 23 | 23 | 33 | 32 |
| *K. pneumoniae* | 2020-331 | 11 | 29 | 26 | 24 | 28 | 21 | 32 | 21 | 31 | 25 | 21 | 30 | 29 | 26 | 25 | 34 | 32 |
| *K. pneumoniae* | 2020-332 | 10 | 29 | 26 | 23 | 27 | 19 | 32 | 19 | 30 | 25 | 21 | 30 | 28 | 26 | 24 | 34 | 32 |
| *K. pneumoniae* | 2020-375 | 9 | 28 | 25 | 24 | 27 | 20 | 32 | 16 | 29 | 25 | 20 | 30 | 30 | 26 | 26 | 33 | 35 |
| *K. oxytoca* | 2016-639 | 11 | 28 | 27 | 25 | 27 | 21 | 32 | 20 | 30 | 25 | 22 | 30 | 29 | 26 | 26 | 34 | 33 |
| *K. oxytoca* | 2016-683 | 9 | 29 | 28 | 26 | 28 | 21 | 30 | 18 | 30 | 24 | 20 | 30 | 29 | 26 | 27 | 35 | 33 |
| *K. oxytoca* | 2016-731 | 16 | 31 | 29 | 29 | 31 | 21 | 33 | 21 | 30 | 27 | 22 | 31 | 30 | 27 | 28 | 36 | 33 |
| *K. oxytoca* | 2016-813 | 0 | 28 | 26 | 24 | 28 | 20 | 30 | 18 | 27 | 24 | 20 | 29 | 29 | 28 | 26 | 34 | 32 |
| *K. oxytoca* | 2016-827 | 11 | 30 | 27 | 26 | 29 | 21 | 32 | 22 | 31 | 26 | 21 | 30 | 30 | 28 | 26 | 34 | 34 |
| *K. oxytoca* | 2016-832 | 11 | 29 | 28 | 25 | 28 | 21 | 31 | 20 | 31 | 26 | 22 | 31 | 30 | 26 | 27 | 34 | 32 |
| *K. oxytoca* | 2016-930 | 0 | 28 | 25 | 22 | 28 | 21 | 32 | 24 | 28 | 26 | 21 | 30 | 29 | 25 | 24 | 31 | 32 |
| *K. oxytoca* | 2016-1028 | 11 | 28 | 28 | 24 | 28 | 20 | 32 | 22 | 30 | 26 | 21 | 29 | 28 | 25 | 26 | 33 | 32 |
| *K. oxytoca* | 2016-1146 | 0 | 28 | 25 | 24 | 28 | 20 | 31 | 22 | 29 | 25 | 22 | 29 | 30 | 26 | 27 | 34 | 34 |
| *K. oxytoca* | 2016-1170 | 12 | 30 | 28 | 26 | 28 | 21 | 31 | 21 | 31 | 26 | 22 | 30 | 29 | 25 | 27 | 32 | 31 |
| *K. oxytoca* | 2016-1244 | 0 | 26 | 27 | 23 | 28 | 20 | 32 | 19 | 27 | 26 | 21 | 30 | 30 | 27 | 25 | 34 | 34 |
| *K. oxytoca* | 2016-1374 | 0 | 30 | 27 | 26 | 29 | 21 | 32 | 21 | 26 | 26 | 22 | 28 | 27 | 26 | 26 | 34 | 32 |
| *K. oxytoca* | 2019-1389 | 0 | 30 | 26 | 26 | 28 | 21 | 32 | 23 | 30 | 26 | 21 | 30 | 28 | 28 | 25 | 33 | 32 |
| *K. oxytoca* | 2019-1435 /1 | 10 | 27 | 27 | 24 | 27 | 20 | 31 | 19 | 29 | 25 | 21 | 29 | 27 | 25 | 26 | 32 | 30 |
| *K. oxytoca* | 2019-1436 | 0 | 31 | 28 | 27 | 30 | 23 | 31 | 21 | 31 | 25 | 21 | 30 | 29 | 27 | 26 | 33 | 32 |
| *K. oxytoca* | 2019-1489 | 0 | 31 | 27 | 26 | 30 | 21 | 32 | 23 | 26 | 29 | 22 | 31 | 30 | 28 | 26 | 34 | 33 |
| *K. oxytoca* | 2019-1641 | 10 | 32 | 29 | 28 | 29 | 19 | 34 | 22 | 30 | 28 | 22 | 29 | 26 | 26 | 26 | 34 | 34 |
| *K. oxytoca* | 2019-1500 | 0 | 30 | 27 | 25 | 30 | 21 | 32 | 21 | 27 | 25 | 21 | 29 | 29 | 27 | 23 | 31 | 33 |
| *K. oxytoca* | 2019-1521 | 0 | 27 | 26 | 23 | 28 | 20 | 32 | 22 | 28 | 25 | 20 | 31 | 29 | 28 | 28 | 34 | 34 |
| *K. oxytoca* | 2019-1793 | 13 | 29 | 27 | 26 | 28 | 21 | 32 | 20 | 31 | 26 | 23 | 31 | 29 | 25 | 27 | 34 | 32 |
| *K. oxytoca* | 2019-1794 | 15 | 30 | 29 | 26 | 30 | 22 | 32 | 21 | 32 | 26 | 23 | 31 | 29 | 26 | 26 | 35 | 33 |
| *K. oxytoca* | 2019-1579 | 10 | 30 | 28 | 25 | 30 | 20 | 32 | 24 | 30 | 26 | 21 | 31 | 28 | 28 | 26 | 33 | 32 |
| *K. oxytoca* | 2019-1907 | 11 | 31 | 28 | 27 | 31 | 19 | 32 | 21 | 30 | 26 | 21 | 31 | 29 | 27 | 30 | 36 | 34 |
| *K. oxytoca* | 2019-1908 | 12 | 29 | 27 | 29 | 32 | 20 | 32 | 21 | 29 | 26 | 22 | 32 | 29 | 28 | 28 | 36 | 34 |
| *K. oxytoca* | 2019-1911 | 10 | 30 | 28 | 26 | 30 | 19 | 33 | 24 | 30 | 27 | 21 | 30 | 30 | 26 | 25 | 35 | 34 |
| *K. oxytoca* | 2019-1954 | 11 | 30 | 27 | 26 | 30 | 19 | 34 | 18 | 32 | 27 | 22 | 31 | 29 | 25 | 28 | 36 | 35 |
| *K. oxytoca* | 2019-2014 | 0 | 31 | 28 | 26 | 31 | 22 | 34 | 25 | 31 | 28 | 23 | 32 | 27 | 26 | 26 | 34 | 35 |
| *K. oxytoca* | 2019-2019 | 0 | 34 | 27 | 25 | 31 | 21 | 34 | 26 | 31 | 27 | 22 | 30 | 26 | 25 | 27 | 34 | 35 |
| *K. oxytoca* | 2019-2016 | 0 | 30 | 28 | 24 | 31 | 20 | 33 | 26 | 29 | 36 | 22 | 31 | 26 | 25 | 27 | 36 | 36 |
| *K. oxytoca* | 2019-2018 | 0 | 31 | 26 | 24 | 30 | 20 | 33 | 26 | 30 | 27 | 23 | 29 | 26 | 26 | 28 | 35 | 33 |
| *K. oxytoca* | 2019-2017 /1 | 0 | 32 | 27 | 25 | 32 | 22 | 34 | 26 | 32 | 28 | 23 | 32 | 26 | 26 | 27 | 34 | 35 |
| *K. oxytoca* | 2019-2022 | 0 | 31 | 27 | 25 | 30 | 21 | 32 | 20 | 32 | 26 | 23 | 31 | 26 | 27 | 25 | 33 | 32 |
| *K. oxytoca* | 2019-2195 | 0 | 32 | 28 | 27 | 31 | 20 | 33 | 22 | 31 | 26 | 22 | 30 | 27 | 28 | 25 | 35 | 34 |
| *K. oxytoca* | 2020-32 | 0 | 27 | 26 | 25 | 30 | 20 | 34 | 21 | 32 | 27 | 22 | 32 | 27 | 27 | 22 | 30 | 32 |
| *K. oxytoca* | 2020-318 | 0 | 31 | 28 | 27 | 29 | 21 | 34 | 23 | 32 | 27 | 22 | 30 | 26 | 26 | 27 | 34 | 34 |
| *K. oxytoca* | 2020-227 | 9 | 32 | 28 | 26 | 31 | 22 | 32 | 22 | 31 | 28 | 21 | 32 | 27 | 26 | 27 | 33 | 34 |
| *K. oxytoca* | 2020-230 | 0 | 29 | 27 | 26 | 31 | 20 | 34 | 23 | 31 | 27 | 22 | 32 | 28 | 27 | 24 | 33 | 35 |
| *K. oxytoca* | 2020-233 | 13 | 32 | 29 | 28 | 31 | 20 | 34 | 18 | 32 | 27 | 22 | 33 | 28 | 27 | 28 | 36 | 36 |
| *K. oxytoca* | 2019-2191 | 0 | 32 | 28 | 27 | 33 | 21 | 33 | 20 | 32 | 27 | 21 | 32 | 30 | 28 | 28 | 36 | 35 |
| *K. oxytoca* | 2019-2197 /2 | 0 | 30 | 27 | 26 | 30 | 21 | 34 | 24 | 32 | 26 | 22 | 32 | 30 | 27 | 28 | 36 | 34 |
| *K. oxytoca* | 2020-317 | 0 | 27 | 24 | 24 | 28 | 20 | 31 | 17 | 28 | 26 | 20 | 30 | 29 | 27 | 25 | 33 | 34 |
| *K. variicola* | 2016-260 | 17 | 29 | 26 | 25 | 30 | 21 | 30 | 20 | 27 | 25 | 20 | 31 | 29 | 26 | 26 | 35 | 32 |
| *K. variicola* | 2016-262 | 18 | 29 | 26 | 26 | 28 | 20 | 29 | 20 | 27 | 25 | 19 | 29 | 28 | 26 | 27 | 33 | 30 |
| *K. variicola* | 2016-501 | 10 | 25 | 23 | 21 | 23 | 20 | 28 | 15 | 28 | 22 | 19 | 27 | 24 | 24 | 23 | 30 | 30 |
| *K. variicola* | 2016-510 | 11 | 28 | 24 | 24 | 25 | 21 | 30 | 17 | 28 | 24 | 20 | 30 | 27 | 25 | 26 | 34 | 33 |
| *K. variicola* | 2016-1065 | 11 | 27 | 25 | 22 | 25 | 20 | 29 | 17 | 25 | 22 | 19 | 29 | 28 | 26 | 26 | 32 | 31 |
| *K. variicola* | 2016-1297 | 9 | 26 | 23 | 22 | 25 | 20 | 29 | 18 | 25 | 23 | 20 | 28 | 25 | 25 | 22 | 30 | 30 |
| *K. variicola* | 2016-1369 | 11 | 29 | 24 | 24 | 26 | 21 | 29 | 18 | 28 | 23 | 20 | 30 | 29 | 27 | 25 | 32 | 31 |
| *K. variicola* | 2016-1370 | 10 | 28 | 23 | 23 | 25 | 20 | 30 | 21 | 31 | 24 | 20 | 29 | 27 | 26 | 25 | 33 | 33 |
| *K. variicola* | 2016-1402 | 9 | 25 | 23 | 21 | 24 | 21 | 28 | 17 | 24/30 | 23 | 18 | 29 | 26 | 25 | 24 | 32 | 32 |
| *K. variicola* | 2019-1417 /1 | 11 | 27 | 24 | 23 | 24 | 19 | 28 | 20 | 28 | 22 | 19 | 30 | 28 | 27 | 26 | 32 | 33 |
| *K. variicola* | 2019-1390 | 0 | 30 | 27 | 26 | 27 | 23 | 32 | 24 | 30 | 24 | 21 | 30 | 27 | 25 | 27 | 32 | 32 |
| *K. variicola* | 2019-1445 /1 | 10 | 30 | 25 | 25 | 26 | 21 | 31 | 22 | 29 | 24 | 20 | 30 | 28 | 27 | 25 | 33 | 32 |
| *K. variicola* | 2019-1461 | 11 | 27 | 25 | 23 | 27 | 19 | 33 | 23 | 30 | 26 | 20 | 30 | 29 | 26 | 25 | 34 | 32 |
| *K. variicola* | 2019-1627 | 18 | 30 | 27 | 26 | 30 | 20 | 31 | 20 | 27 | 25 | 20 | 30 | 27 | 28 | 27 | 36 | 32 |
| *K. variicola* | 2019-1762 | 9 | 27 | 23 | 21 | 25 | 20 | 31 | 20 | 30 | 24 | 20 | 30 | 29 | 25 | 23 | 34 | 33 |
| *K. variicola* | 2019-1580 | 10 | 29 | 24 | 23 | 26 | 21 | 31 | 18 | 29 | 21 | 19 | 29 | 27 | 24 | 23 | 32 | 30 |
| *K. variicola* | 2019-1848 | 9 | 29 | 24 | 22 | 25 | 19 | 30 | 27 | 31 | 25 | 20 | 32 | 26 | 25 | 25 | 36 | 34 |
| *K. variicola* | 2019-1904 | 23 | 28 | 25 | 23 | 26 | 21 | 30 | 22 | 30 | 25 | 21 | 27 | 24 | 26 | 23 | 34 | 32 |
| *K. variicola* | 2019-2013 | 19 | 28 | 27 | 27 | 30 | 20 | 34 | 23 | 32 | 25 | 21 | 31 | 29 | 26 | 29 | 36 | 32 |
| *K. variicola* | 2019-2057 | 8 | 30 | 25 | 23 | 29 | 21 | 32 | 18 | 31 | 26 | 21 | 30 | 25 | 25 | 24 | 33 | 32 |
| *K. variicola* | 2019-2058 | 12 | 29 | 24 | 25 | 28 | 20 | 33 | 22 | 30 | 27 | 20 | 31 | 28 | 26 | 26 | 36 | 35 |
| *K. variicola* | 2019-2190 | 16 | 30 | 26 | 29 | 31 | 19 | 35 | 22 | 33 | 28 | 23 | 32 | 29 | 26 | 29 | 36 | 35 |
| *K. variicola* | 2019-2193 | 15 | 25 | 25 | 28 | 31 | 19 | 34 | 19 | 32 | 29 | 22 | 32 | 28 | 27 | 29 | 34 | 32 |
| *K. variicola* | 2019-2194 | 16 | 31 | 26 | 29 | 32 | 19 | 33 | 20 | 32 | 29 | 22 | 32 | 30 | 29 | 29 | 36 | 32 |
| *K. variicola* | 2019-2063 /1 | 10 | 30 | 27 | 27 | 29 | 21 | 33 | 19 | 30 | 25 | 22 | 30 | 28 | 25 | 25 | 34 | 34 |
| *K. variicola* | 2019-2059 | 10 | 28 | 24 | 22 | 26 | 20 | 31 | 20 | 30 | 23 | 19 | 29 | 27 | 24 | 23 | 34 | 33 |
| *K. variicola* | 2019-2117 | 10 | 28 | 25 | 22 | 25 | 21 | 31 | 18 | 30 | 23 | 19 | 30 | 29 | 25 | 25 | 35 | 35 |
| *K. variicola* | 2019-2061 | 9 | 26 | 24 | 23 | 27 | 19 | 32 | 18 | 29 | 26 | 20 | 30 | 30 | 27 | 26 | 35 | 32 |
| *K. variicola* | 2020-374 | 10 | 29 | 24 | 23 | 27 | 21 | 32 | 22 | 29 | 25 | 21 | 30 | 29 | 24 | 24 | 36 | 32 |
| *K. variicola* | 2019-2062 | 0 | 28 | 26 | 24 | 27 | 20 | 31 | 18 | 29 | 25 | 21 | 31 | 28 | 27 | 26 | 35 | 35 |
| *K. variicola* | 2020-371 /1 | 13 | 28 | 24 | 24 | 28 | 19 | 34 | 21 | 29 | 26 | 21 | 30 | 30 | 27 | 27 | 36 | 34 |
| *K. variicola* | 2020-312 /1 | 11 | 31 | 25 | 25 | 29 | 21 | 33 | 21 | 31 | 24 | 20 | 30 | 29 | 27 | 25 | 36 | 34 |
| *K. variicola* | 2019-2197 /1 | 0 | 32 | 27 | 24 | 28 | 21 | 33 | 21 | 31 | 26 | 21 | 31 | 29 | 25 | 24 | 33 | 34 |
| *K. aerogenes* | 2019-1885 | 0 | 27 | 12 | 24 | 27 | 19 | 34 | 13 | 30 | 25 | 21 | 28 | 26 | 0 | 25 | 33 | 35 |
| *R. ornithinolyticia* | 2016-1035 | 0 | 29 | 28 | 26 | 29 | 21 | 31 | 22 | 29 | 25 | 20 | 29 | 29 | 26 | 27 | 33 | 32 |
| *R. ornithinolyticia* | 2016-1176 | 0 | 28 | 28 | 25 | 28 | 21 | 31 | 22 | 30 | 24 | 21 | 30 | 28 | 26 | 28 | 33 | 34 |
| *R. ornithinolyticia* | 2016-1199 | 0 | 26 | 27 | 24 | 27 | 21 | 32 | 24 | 33 | 24 | 20 | 30 | 27 | 25 | 25 | 32 | 34 |
| *R. ornithinolyticia* | 2016-1239 | 0 | 27 | 28 | 25 | 28 | 20 | 32 | 21 | 29 | 25 | 21 | 30 | 29 | 25 | 27 | 33 | 33 |
| *R. ornithinolyticia* | 2016-1254 | 13 | 28 | 28 | 27 | 28 | 22 | 33 | 23 | 33 | 26 | 22 | 31 | 27 | 26 | 28 | 34 | 33 |
| *R. ornithinolyticia* | 2016-538 | 12 | 29 | 28 | 28 | 26 | 23 | 32 | 22 | 30 | 25 | 20 | 30 | 28 | 25 | 26 | 33 | 32 |
| *R. ornithinolyticia* | 2016-651 | 10 | 30 | 29 | 26 | 27 | 21 | 34 | 22 | 31 | 25 | 21 | 30 | 29 | 26 | 26 | 34 | 33 |
| *R. ornithinolyticia* | 2016-682 | 9 | 28 | 27 | 26 | 27 | 23 | 32 | 24 | 32 | 27 | 21 | 30 | 27 | 26 | 26 | 32 | 32 |
| *R. ornithinolyticia* | 2016-738 | 10 | 27 | 27 | 25 | 29 | 22 | 32 | 23 | 30 | 26 | 21 | 29 | 28 | 26 | 26 | 33 | 32 |
| *R. ornithinolyticia* | 2016-881 | 11 | 26 | 27 | 26 | 28 | 21 | 31 | 22 | 31 | 26 | 20 | 30 | 27 | 27 | 27 | 34 | 32 |
| *R. ornithinolyticia* | 2016-923 | 0 | 20 | 24 | 23 | 26 | 21 | 30 | 19 | 28 | 23 | 19 | 29 | 26 | 23 | 24 | 31 | 30 |
| *R. ornithinolyticia* | 2019-1387 | 11 | 28 | 28 | 25 | 30 | 22 | 33 | 24 | 31 | 26 | 21 | 31 | 28 | 27 | 27 | 33 | 33 |
| *R. ornithinolyticia* | 2019-1391 | 0 | 26 | 27 | 25 | 29 | 21 | 33 | 23 | 31 | 26 | 21 | 32 | 29 | 28 | 28 | 34 | 32 |
| *R. ornithinolyticia* | 2019-1445/2 | 0 | 25 | 28 | 24 | 27 | 21 | 31 | 20 | 28 | 24 | 20 | 30 | 27 | 26 | 25 | 33 | 32 |
| *R. ornithinolyticia* | 2019-1488 | 0 | 27 | 26 | 25 | 27 | 21 | 31 | 22 | 31 | 25 | 20 | 31 | 28 | 26 | 27 | 33 | 33 |
| *R. ornithinolyticia* | 2019-1494 | 0 | 24 | 27 | 25 | 28 | 21 | 32 | 22 | 32 | 26 | 22 | 30 | 29 | 26 | 27 | 34 | 33 |
| *R. ornithinolyticia* | 2019-1495 | 11 | 26 | 27 | 25 | 28 | 21 | 33 | 24 | 29 | 26 | 20 | 30 | 29 | 26 | 28 | 34 | 32 |
| *R. ornithinolyticia* | 2019-1498 | 0 | 25 | 28 | 25 | 27 | 20 | 34 | 22 | 32 | 26 | 21 | 31 | 26 | 24 | 25 | 34 | 31 |
| *R. ornithinolyticia* | 2019-1583 | 0 | 27 | 28 | 25 | 28 | 22 | 32 | 23 | 31 | 24 | 20 | 32 | 28 | 26 | 26 | 32 | 31 |
| *R. ornithinolyticia* | 2019-1637 | 13 | 30 | 28 | 29 | 31 | 21 | 34 | 25 | 31 | 29 | 22 | 32 | 28 | 26 | 29 | 35 | 33 |
| *R. ornithinolyticia* | 2019-1639 | 14 | 30 | 29 | 29 | 32 | 21 | 35 | 26 | 34 | 28 | 23 | 33 | 30 | 29 | 30 | 37 | 33 |
| *R. ornithinolyticia* | 2019-1642 | 16 | 29 | 28 | 28 | 30 | 23 | 34 | 26 | 33 | 26 | 22 | 31 | 30 | 29 | 28 | 34 | 32 |
| *R. ornithinolyticia* | 2019-1717 | 19 | 28 | 29 | 26 | 30 | 22 | 33 | 21 | 31 | 26 | 21 | 31 | 30 | 26 | 27 | 35 | 33 |
| *R. ornithinolyticia* | 2019-1724 | 0 | 21 | 28 | 25 | 28 | 22 | 33 | 24 | 32 | 24 | 21 | 31 | 28 | 26 | 30 | 36 | 35 |
| *R. ornithinolyticia* | 2019-1725 | 0 | 23 | 28 | 26 | 29 | 22 | 33 | 24 | 31 | 25 | 21 | 30 | 22 | 23/22 | 24 | 31 | 31 |
| *R. ornithinolyticia* | 2019-1786 | 9 | 28 | 27 | 24 | 29 | 21 | 32 | 23 | 28 | 26 | 21 | 31 | 28 | 26 | 26 | 34 | 32 |
| *R. ornithinolyticia* | 2019-1791/2 | 0 | 24 | 27 | 24 | 27 | 20 | 31 | 20 | 30 | 24 | 21 | 29 | 27 | 25 | 26 | 32 | 32 |
| *R. ornithinolyticia* | 2019-1842 | 0 | 25 | 28 | 24 | 28 | 21 | 33 | 20 | 32 | 25 | 22 | 29 | 26 | 26 | 24 | 33 | 32 |
| *R. ornithinolyticia* | 2019-1899 | 0 | 32 | 28 | 26 | 31 | 22 | 35 | 24 | 32 | 26 | 21 | 32 | 25 | 26 | 26 | 34 | 36 |
| *R. ornithinolyticia* | 2019-1900 | 0 | 32 | 29 | 26 | 32 | 23 | 35 | 25 | 34 | 27 | 23 | 30 | 28 | 26 | 26 | 35 | 32 |
| *R. ornithinolyticia* | 2019-1901 | 0 | 21 | 26 | 24 | 27 | 21 | 33 | 24 | 32 | 26 | 22 | 32 | 29 | 28 | 27 | 36 | 36 |
| *R. ornithinolyticia* | 2019-1905 | 0 | 31 | 28 | 27 | 31 | 22 | 33 | 22 | 33 | 26 | 22 | 32 | 28 | 27 | 27 | 34 | 34 |
| *R. ornithinolyticia* | 2019-2021 | 12 | 30 | 29 | 29 | 32 | 22 | 35 | 27 | 33 | 29 | 22 | 32 | 30 | 29 | 29 | 36 | 36 |
| *R. ornithinolyticia* | 2019-2189 | 0 | 27 | 27 | 24 | 28 | 21 | 32 | 21 | 31 | 24 | 20 | 32 | 27 | 24 | 26 | 33 | 34 |
| *R. ornithinolyticia* | 2020-232 | 9 | 27 | 28 | 23 | 28 | 21 | 31 | 20 | 31 | 25 | 21 | 30 | 27 | 24 | 25 | 33 | 32 |
| *R. ornithinolyticia* | 2020-271 | 13 | 29 | 29 | 28 | 31 | 21 | 33 | 23 | 32 | 27 | 21 | 32 | 31 | 28 | 29 | 36 | 36 |
| *R. ornithinolyticia* | 2020-273 | 0 | 31 | 29 | 27 | 30 | 21 | 33 | 22 | 33 | 27 | 21 | 32 | 30 | 26 | 27 | 34 | 36 |
| *R. ornithinolyticia* | 2020-278 | 20 | 32 | 29 | 30 | 29 | 22 | 35 | 27 | 34 | 29 | 22 | 32 | 31 | 27 | 29 | 37 | 37 |
| *R. planticola* | 2016-1197 | 0 | 24 | 27 | 24 | 27 | 21 | 32 | 23 | 33 | 24 | 20 | 29 | 24 | 23 | 26 | 31 | 33 |
| *R. planticola* | 2016-1256 | 0 | 25 | 27 | 24 | 26 | 21 | 32 | 22 | 30 | 25 | 21 | 30 | 29 | 28 | 26 | 34 | 33 |
| *R. planticola* | 2019-2012 | 0 | 29 | 27 | 26 | 30 | 23 | 35 | 22 | 33 | 25 | 21 | 32 | 27 | 27 | 26 | 35 | 34 |
| *R. planticola* | 2020-371/2 | 0 | 27 | 26 | 26 | 28 | 22 | 33 | 24 | 33 | 25 | 23 | 29 | 27 | 24 | 25 | 33 | 33 |
| *R. planticola* | 2020-324 | 0 | 29 | 28 | 27 | 31 | 22 | 32 | 22 | 31 | 25 | 22 | 31 | 31 | 26 | 27 | 36 | 35 |
| *R. planticola* | 2019-2011/2 | 0 | 27 | 27 | 26 | 28 | 22 | 34 | 23 | 33 | 24 | 21 | 32 | 28 | 25 | 26 | 33 | 33 |
| *R. planticola* | 2016-1034 | 0 | 25 | 27 | 24 | 26 | 21 | 32 | 22 | 31 | 24 | 19 | 29 | 26 | 25 | 25 | 32 | 32 |
| *R. planticola* | 2019-1493/1 | 0 | 23 | 26 | 24 | 28 | 22 | 32 | 23 | 32 | 25 | 20 | 31 | 27 | 26 | 26 | 31 | 33 |
| *R. planticola* | 2016-1378 | 0 | 29 | 28 | 26 | 29 | 22 | 32 | 22 | 32 | 25 | 21 | 29 | 26 | 26 | 26 | 32 | 30 |
| *R. planticola* | 2019-2201 | 0 | 25 | 28 | 25 | 29 | 21 | 33 | 20 | 32 | 26 | 22 | 30 | 29 | 26 | 25 | 35 | 34 |
| *R. planticola* | 2019-1498 /2 | 0 | 22 | 26 | 23 | 26 | 21 | 31 | 21 | 29 | 25 | 21 | 30 | 26 | 26 | 25 | 32 | 32 |
| *R. planticola* | 2016-1253 | 0 | 25 | 26 | 24 | 27 | 22 | 32 | 22 | 31 | 26 | 21 | 30 | 27 | 25 | 26 | 33 | 32 |
| *R. planticola* | 2016-1329 | 0 | 25 | 28 | 24 | 27 | 20 | 30 | 20 | 28 | 23 | 20 | 30 | 26 | 25 | 24 | 30 | 30 |

Abbreviations: AMP: Ampicillin, MEL: Mecillinam, AMC: Amoxicillin-clavulanic acid, TZP: Piperacillin-Tazobactam, CHL: Chloramphenicol, GEN: Gentamicin, CIP: Ciprofloxacin, NIT: Nitrofurantoin, SXT: Trimethoprim-sulfamethoxazole, TET: Tetracycline, TGC: Tigecycline, CTX: Cefotaxime, CAZ: Ceftazidime, FOX: Cefoxitin, CXM: Cefuroxime, ATM: Aztreonam, MEM: Meropenem
